# The Use of Plant Viral Nanoparticles in Cancer Biotherapy—A Review

**DOI:** 10.3390/v17020218

**Published:** 2025-02-01

**Authors:** Mamorake Donty Komane, Prudence Ngalula Kayoka-Kabongo, Daria Anna Rutkowska

**Affiliations:** 1 Department of Agriculture and Animal Health, College of Agriculture and Environmental Sciences, University of South Africa, Science Campus, Private Bag X6 Florida 1710, Pretoria 0002, South Africa; 56749988@mylife.unisa.ac.za (M.D.K.); kabonpnk@unisa.ac.za (P.N.K.-K.); 2 Advanced Agriculture and Food Cluster, Council for Scientific and Industrial Research, P.O. Box 395, Pretoria 0001, South Africa

**Keywords:** plant viral nanoparticles, cancer biotherapy, drug delivery, virus-like particles, cancer vaccines, tumor targeting, cancer immunotherapy, targeted delivery, imaging agents, plant viruses

## Abstract

Cancer is a major global health problem that poses significant challenges. Conventional cancer therapies often have severe side effects, necessitating the development of novel therapeutic approaches that are more effective and less toxic. The utilization of plant viral nanoparticles is one of the more promising strategies for cancer biotherapy. Plant viral nanoparticles exhibit advantageous properties, including safety, high stability, rapid production and scalability, biocompatibility and biodegradability, structural uniformity, inherent immunogenicity, ease of modification and high update efficacy as well as lower cost implications, making them attractive vehicles for health applications. Various studies have demonstrated the efficacy of plant viral nanoparticles in targeted therapeutic drug/molecule delivery, tumor imaging and immunotherapy, highlighting their potential as a versatile platform for cancer biotherapy. The drawbacks of plant viral nanoparticles include their perceived ability to induce a hypersensitive/allergic immune response, non-well-defined regulatory approval processes as well as the reluctance of pharmaceutical companies to adapt their manufacturing processes to facilitate plant-based expression. This review discusses applications of plant virus-derived nanoparticles in cancer therapeutics and prospects for translating these findings into clinical practice.

## 1. Introduction

Cancer remains a significant global health problem, accounting for approximately 19.96 million cases in 2022 [1], despite advancements in conventional treatments, such as chemotherapy, surgery and radiation [2]. These treatments frequently have limitations, such as nonspecific targeting, severe side effects and drug resistance [3]. Thus, there is a need for innovative therapeutic approaches that can combat these challenges. Nanotechnology has opened a new platform in cancer biotherapy, with nanoparticles that allow for targeted drug delivery, enhanced therapeutic efficacy and reduced systemic toxicity [4]. Viral nanoparticles (VNPs) are virus-based nanoparticles that may be bacteriophages or plant or mammalian viruses, among others, and may be infectious or non-infectious [5]. Plant virus-based nanoparticles are among the different types of nanoparticles explored for cancer therapy and have gained popularity due to their biocompatibility, low toxicity as well as reduced ecological impact [5]. Plant viral nanoparticles (PVNPs) are naturally occurring nanostructures generated when plant viral coat proteins self-assemble into highly ordered, homogeneous geometric structures, such as spheres, rods or filaments [6]. The unique structure of these VNPs offers promising delivery vehicle capability, including the potential to contain a variety of therapeutic payloads [7]. Besides their homogenous and stable structure, PVNPs can be genetically or chemically modified to enhance their targeting and therapeutic capabilities [8,9]. Plant-based synthesis is scalable, cost-effective and environmentally sustainable compared to the generation of synthetic or animal-derived nanoparticles [9,10,11].

Plant viral nanoparticles have a wide range of applications in cancer biotherapy, from drug delivery to cancer vaccines to enzyme-based delivery therapies [12,13,14,15]. PVNPs can be designed to deliver chemotherapeutic agents to cancer cells, thus increasing the amount of drug at the tumor site and reducing side effects [10,12]. PVNPs can also serve as cancer vaccine platforms by exhibiting tumor antigens and inducing antitumor immune responses [13]. Furthermore, PVNPs have also made promising advances in enzyme delivery, extracellular matrix degradation and modification of the tumor microenvironment (TME) to improve drug penetration [16]. Plant viral nanoparticles have also been identified as potential useful tools for medical imaging and therapeutics. These targeting characteristics are promising, but challenges still exist in heterogeneity for efficient targeting, immune clearance and reproducible treatment effects [17]. Here, we review the status of PVNPs in cancer biotherapy.

## 2. Materials and Methods

**Figure 1:**
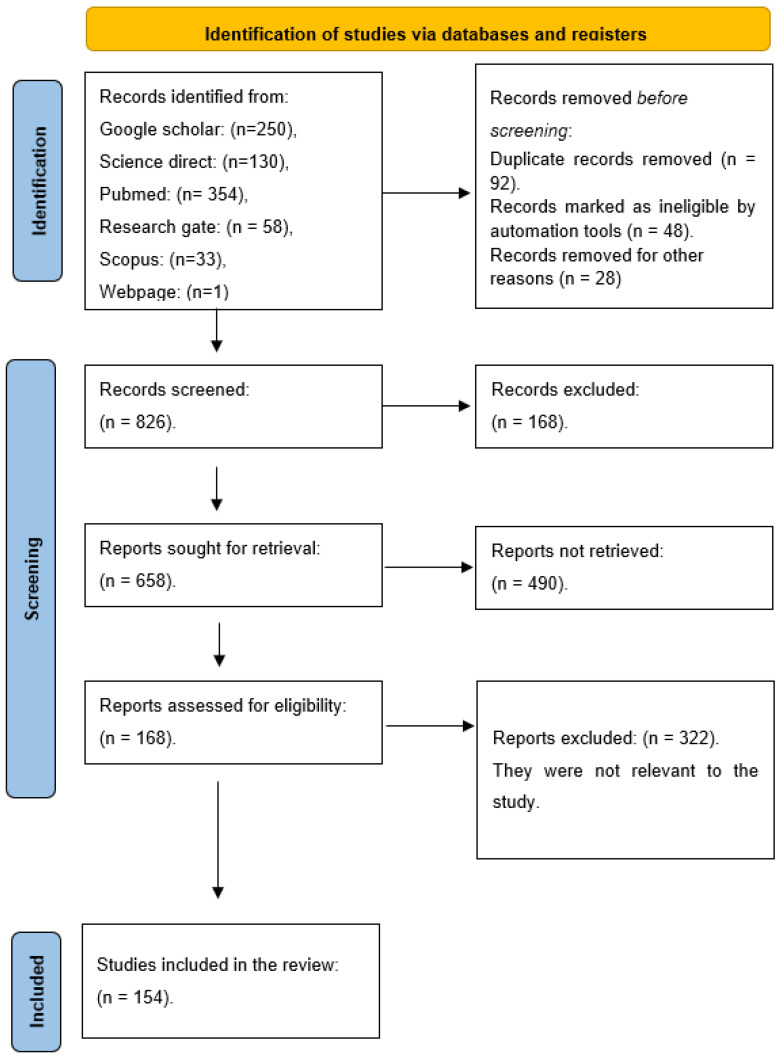
Illustration of the PRISMA flow diagram outlining the process of identifying, screening and selecting articles for inclusion in the review.

A systematic review was conducted to evaluate the application of plant viral nanoparticles in cancer biotherapy, adhering to the guidelines outlined in the Preferred Reporting Items for Systematic Reviews and Meta-Analyses (PRISMA) framework [18]. The methodology followed is illustrated in Figure 1.

### 2.1. Eligibility Criteria

The inclusion criteria focused on peer-reviewed journal articles, review articles, research papers and book chapters written in English only from the year 1998 to 2024. It also included studies reporting preclinical or clinical outcomes in cancer biotherapy. Articles that were not written in English, duplicates, not relevant to PVNPs, commentaries, opinion pieces without original data and noncancer applications as well as all articles prior to 1998 were excluded.

### 2.2. Search Strategy

A comprehensive literature search was performed using multiple electronic databases, including PubMed, Google Scholar, Research Gate, Scopus and Science Direct, ensuring comprehensive coverage of research. Search strategies combined keywords such as plant viral nanoparticles, virus-like particles, cancer therapeutics, cancer vaccines and cancer immunotherapy. Our focus was on plant viral expressed systems, as there are also other systems.

### 2.3. Risk of Bias Assessment

This review focused on original research articles, review papers, book chapters and conference papers. To maintain the quality of the paper, all duplicates were checked thoroughly, article abstracts were included to guarantee academic literature relevance and quality, and each paper was carefully evaluated.

## 3. Results and Discussion

### 3.1. Structure of Plant Viral Nanoparticles

Plant viral nanoparticles (PVNPs), derived from plant viruses such as Cowpea mosaic virus (CPMV), Tobacco mosaic virus (TMV) and Papaya mosaic virus (PapMV), among others, provide a wide range of applications for cancer therapy [19]. PVNPs are suitable for such applications as they are non-infectious to humans, unlike animal- or human-derived viral nanoparticles [20]. Their plant-based nature ensures that they are non-pathogenic and less prone to triggering harmful immune responses, which is an important factor in cancer treatment [21].

**Figure 2:**
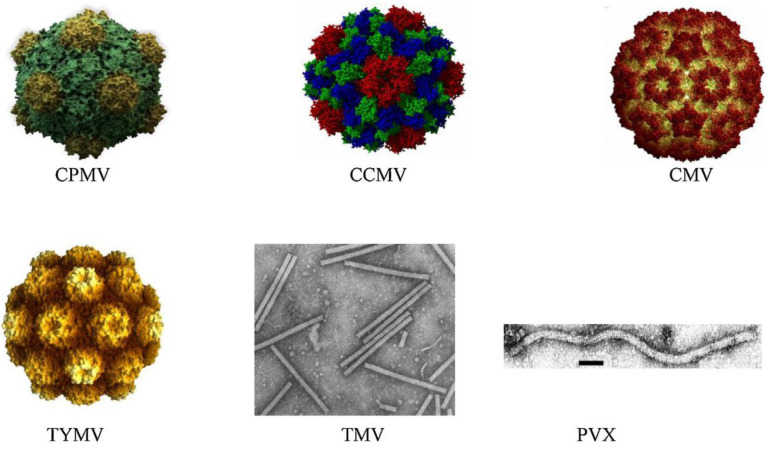
Schematic representation of plant viruses with capsid structures of various shapes (Icosahedral Cowpea mosaic virus (CPMV), Cowpea chlorotic mottle virus (CCMV), Cucumber mosaic virus (CMV), Turnip yellow mosaic virus (TYMV), Rod-shaped Tobacco mosaic virus (TMV), Potato virus X (PVX)) from reference [23] with permission.

The plant viruses used to construct PVNPs are carefully selected because each virus has unique structural characteristics lending itself to diverse therapeutic uses [22]. Figure 2 illustrates plant viruses with capsids of various shapes [23]. Cowpea mosaic virus, one of the most researched plant viruses in the field of nanotechnology, generates icosahedral particles approximately 30 nm in diameter [7,9,11]. Such particles are very robust and can tolerate extensive chemical modification [11]. CPMV is particularly well suited for drug delivery since it can encapsulate medicines or other therapeutic materials within its capsid or can be functionalized with targeting ligands on its surface [24]. According to [24], CPMV can be loaded with chemotherapeutic drugs, such as doxorubicin, thus enhancing the targeting and therapeutic efficacy of anticancer agents [25]. In a vaccine approach, initial research showed that injecting CPMV near tumors prompts the immune system to recognize the particles as foreign, drawing immune cells to the area to better target and destroy cancer cells [13]. Recently, researchers synthesized CPMV viral nanoparticle vaccines against the S100A9 calcium-binding protein A9, also known as migration inhibitory factor-related protein 14 or calgranulin B, which is encoded by the S1009 gene [26,27]. This calcium (Ca^2+^)-binding protein is upregulated in various cancer types [28]. These vaccines reduced the levels of S100A9 within the lungs and sera of mice and resulted in reduced tumor seeding and metastasis [29].

Tobacco mosaic virus, on the other hand, has a unique rod shape that is 18 nm wide and 300 nm long, with specific structural advantages [30,31]. Its elongated shape provides a larger surface area that allows for the attachment of larger amounts of therapeutic cargo or targeting ligands [24]. The outer part of TMV can be modified to load cytotoxic agents or peptides that will bind to cancer cells [32]. Researchers have successfully modified TMV to present cancer-targeting peptides, such as RGD peptides, which interact with integrins that are overexpressed in tumor cells [33,34]. This targeting method increases the specificity of the nanoparticle delivery and decreases off-target reactions, leading to less toxicity to surrounding healthy tissues [35].

Papaya mosaic virus is another filamentous virus that is notable for its versatility and ability to deliver therapeutics [7]. PapMV produces nanoparticles of approximately 15 nm in diameter and 100 nm in length, optimal dimensions for performing surface modifications with targeting capability [36]. PapMV has recently been a popular candidate for drug delivery systems designed toward the targeting of melanoma cancer cells [37]. Additionally, the potential use of PapMV nanoparticles has also been investigated as a vector in cancer vaccinations, given their potential to be engineered to display tumor-associated antigens and induce an immune response against malignant cells [38].

### 3.2. Engineering of Plant Viral Nanoparticles

**Figure 3:**
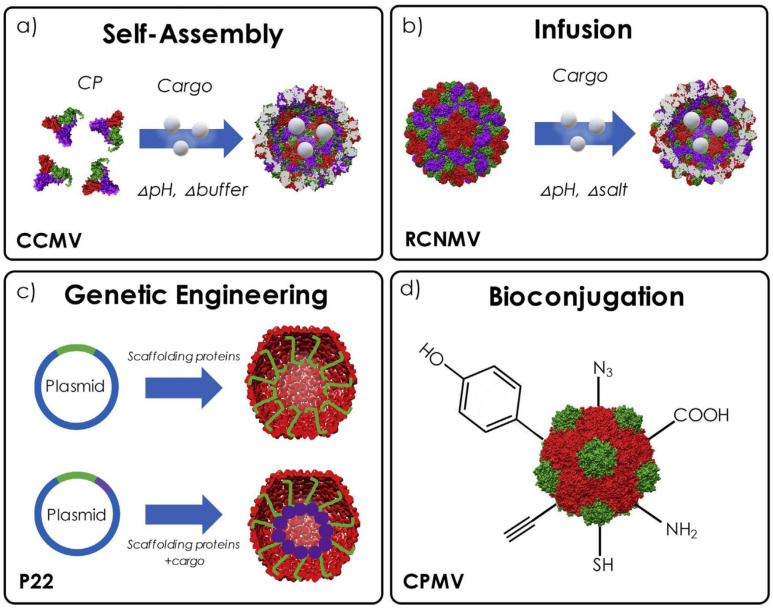
Illustrations of various approaches to loading cargo into VLPs, including (**a**) utilizing pH and buffer condition adjustments to allow for self-assembly around the cargo (demonstrated with CCMV); (**b**) infusing cargo into RCNMV through modifications in pH and salt concentrations; (**c**) applying genetic engineering to attach scaffolding proteins to encapsulate drugs within P22 and (**d**) using bioconjugation to attach cargo to CPMV by targeting residues on its external surface. Permission was obtained from the publisher of [7].

Plant viral nanoparticles can be designed to carry different types of payloads, such as chemotherapeutic compounds and imaging agents, amongst others [6]. By adding specific ligands on their surface, the tumor-targeting capability of these nanoparticles can be improved, therefore increasing the effectiveness of cancer treatment while reducing side effects on healthy tissues [17,22,39]. In addition, PVNPs can be designed to respond to specific stimuli, such as pH changes or enzymatic activity in the tumor microenvironment, allowing for the controlled release of therapeutic agents [40]. The engineering of PVNPs involves manipulating the interior, exterior and interfaces between coat protein (CP) subunits of plant viruses [41]. Virus-like particles (VLPs) are a subset of viral nanoparticles [9]. They are composed entirely of viral structural proteins and lack the genomic components [42,43]. Plant-expressed VLPs, like PVNPs, can be engineered to display ligands or antibodies that bind to receptors, such as platelet-derived growth factor receptor alpha (PDGRα), which are overexpressed on cancer cells [44], allowing for targeted drug delivery or immune activation against cancer cells [45]. Figure 3 depicts the various approaches for loading cargo into VLPs [7]. Some of these approaches can also be used to engineer PVNPs.

Genetic modification involves altering the viral protein sequence to display specific proteins and peptides on the nanoparticle surface [46]. For example, Hovlid et al. [47] genetically displayed Arginyl-glycyl-aspartic acid peptide (RGD peptide) sequences of human adenovirus type 2 on the CPMV coat protein to enhance the ability of nanoparticles (NP) to target integrins involved in cancer cell adhesion. Chemical modification techniques include surface and interior alterations. Surface modification can be achieved through methods such as N-hydroxysuccinimide (NHS) acylation and CU (I)-catalyzed azide-alkyne cycloaddition (CuAAC) that are used to attach cyclic RGD peptides to the viral surface, improving the NP’s targeting capabilities [47,48]. Interior modifications have been demonstrated with empty Cowpea mosaic virus (eCPMV) particles. For example, conjugated fluorophores, biotin affinity tags, polyethyleneglycol and peptides were conjugated to reactive cysteines within the particles to allow for the encapsulation of molecules while maintaining the integrity of the outer surface, potentially improving drug delivery capabilities [49].

For medical imaging applications, VNPs were labelled with near-infrared fluorophores [50]. The fluorophores were bioconjugated to sesbania mosaic virus (SeMV) using reactive lysine-N-hydroxysuccinimide ester and cysteine-maleimide chemistries, and these conjugated SeMV VLPs were internalized into MDA-MB-231 breast cancer cells [51]. Bioconjugated SeMV VLPs could also enter cervical and hepatic cancer cell lines [51]. Similarly, both CPMV and eCPMV were studied for tumor targeting and optical imaging capabilities in vitro and in vivo by modifying these NPs through interior engineering with near-infrared fluorophores through cysteine-specific conjugation chemistry [49]. This modification enabled the creation of multifunctional NPs suitable for various applications, including drug delivery, targeted binding and imaging [52].

### 3.3. Targeting Viral Nanoparticles to Cancer Cells

Molecules that are frequently employed to improve the targeting ability of PVNP-based cancer therapies include peptides, antibodies and aptamers [53]. For example, RGD peptides are widely used to improve the specificity of PVNPs as they target integrins that are overexpressed in multiple cancer types, including breast, lung and colon cancer [10,33]. Similarly, epidermal growth factor receptor (anti-EGFR) antibodies have been linked to PVNPs to target cancer cells with high amounts of EGFR to increase the effectiveness of drug delivery [54]. A study revealed successful targeting of hepatocellular carcinoma using adenovirus nanoparticles functionalized with anti-GPC3 single-chain variable fragments (scFv) [55]. The potential for plant virus-based VLPs to be modified for specific targeting combined with their biodegradability and biocompatibility make them good candidates for future cancer therapy approaches [56]. Examples of plant virus-derived VLPs in targeted cancer biotherapy include potato virus X (PVX) VLPs, which have been conjugated with Herceptin (Trastuzumab) to target HER2-positive breast cancer cells [57]. This bioconjugation was achieved using EDC/sulfo-N-hydroxysuccinimide in a two-step protocol, and the resulting PVX–Herceptin showed efficacy in inducing apoptosis in HER2-positive cancer cell lines compared to Herceptin alone [57,58].

The AS1411 aptamer, which is a short strand of deoxyribonucleic acid (DNA), has been effective in targeting nucleolin, a protein found on the surface of cancer cells, increasing the uptake of metal nanoparticles such as gold (AuNP) [59]. The use of lectins as a means of targeting viral nanoparticles is also being explored [60].

#### Lectins in Cancer Therapy

Lectins are a broad class of proteins characterized by their ability to bind specific carbohydrates and can be utilized in various therapeutic applications such as targeted cancer therapy [60]. Studies have demonstrated the ability of PVNPs modified with lectins to increase the affinity of these particles toward malignant cells [61]. This affinity emanates from lectins’ distinct carbohydrate-binding traits that allow for them to preferentially interact with glycan structures that are frequently overexpressed on the surface of cancer cells [62,63,64]. Because of this unique property, lectins may be suitable to enable targeted delivery of drugs.

Recent studies demonstrate the use of Griffonia simplicifolia lectin (GSL) [65] and wheat germ agglutinin (WGA) [66,67] for the targeting of glycans on various tumors [68,69]. It has also been reported that certain glycans are often aberrantly expressed in tumor cells and can be used as a biological marker for the targeted administration of drugs [70]. Galanthus nivalis agglutinin (GNA) has a high affinity for terminal mannose residues, which are frequently overexpressed on the surface of various cancer cells [71]. The lectins have an inherent ability to differentiate between and attach to certain sugar moieties found on the surface of targeted cells [65]. Therefore, further research is required to evaluate the efficacy and safety of this targeting approach in preclinical models. 

The combination of lectins and VLPs offers a unique approach to enhance targeting and therapeutic efficacy. For example, CPMV VLPs have been conjugated with the plant lectin concanavalin A (Con A) to enhance the targeting and uptake in cancer cells that overexpress mannose receptors [6,72]. Another example includes TMV VLPs that were functionalized with wheat germ agglutinin (WGA) to improve their binding to sialic acid-rich cancer cell surfaces [72,73]. Some plant virus VLPs, including Physalis mottle virus (PhMV) VLPs, have shown uptake by cancer cells due to their potential to recognize altered glycosylation patterns on tumor cell surfaces [74]. This natural affinity can be enhanced by conjugating other lectins to the surface of these VLPs. The ability to engineer VLPs with lectins allows for a tailored targeting of multiple types of cancer, thus improving therapeutic efficacy whilst minimizing side effects [69].

### 3.4. Plant Viral Nanoparticles as Delivery Vehicles in Cancer

#### 3.4.1. Delivery of Cancer Drugs

Plant viral nanoparticles have been investigated as a drug delivery system for cancer chemotherapy. These nanoparticles can be loaded with various chemotherapeutic agents, including hydrophobic drugs, and have shown better tumor-targeting capabilities along with improved pharmacokinetic profiles [75,76]. For example, CPMV nanoparticles have been used for the encapsulation of the chemotherapeutic drug doxorubicin, resulting in higher accumulation of the drug in tumors and reduced toxicity to healthy tissues [11,77].

Drugs can be encapsulated within the hollow capsids of plant viral nanoparticles or can be directly conjugated to their interior surface [31]. This is particularly beneficial for drugs that have low stability or solubility. For example, TMV has encapsulated and delivered cisplatin, an anticancer drug, which prevents the drug from degrading before it reaches the tumor site [78]. The TMV nanoparticle protects it from early release into the bloodstream and allows for a more regulated and prolonged delivery of the medication into the cancer cells [31]. This kind of encapsulation greatly increases the effectiveness of the drug and reduces undesirable side effects, such as toxicity in healthy tissues [79].

#### 3.4.2. Delivery of Therapeutic Genes/Nucleic Acids

Plant viral nanoparticles have emerged as promising vehicles for therapeutic gene and nucleic acid delivery in cancer biotherapy. Studies revealed that PVNPs can efficiently encapsulate and protect nucleic acid cargos, including siRNA, miRNA and plasmid DNA against enzymatic degradation and fast clearance, which are major problems in nucleic acid therapeutics [80,81]. Unique surface features allow for high loading capacity for nucleic acids, avoidance of the immune system and targeting of cancerous cells [82]. It is also possible to engineer PVNPs for improved cellular uptake, endosomal escape and controlled release of the therapeutic payload [83,84]. Cowpea chlorotic mottle virus (CCMV) VLPs were shown to deliver genetic materials directly to mammalian cells [85]. This extended the use of plant viruses to deliver genetic material to mammalian cells and opened new possibilities for targeted gene delivery in cancer [86]. With continuous development in the fields of surface engineering and targeted delivery mechanisms, PVNPs continue to advance as a viable approach to effective nucleic acid-based tumor therapy [87].

#### 3.4.3. Delivery of Therapeutic Enzymes, Proteins and Peptides

Plant viral nanoparticles have a potential role in the delivery of enzymes, proteins and peptides. These nanoparticles can be engineered to present functional amino acids or small peptides on their surface [88,89]. For example, SpyTag/SpyCatcher technology has been applied for the covalent attachment of enzymes, such as Trichoderma reesei endoglucanase Cel12A to Potato virus X (PVX) nanoparticles, thus showing its potential in enzyme immobilization and delivery [88,89]. Cel12A is an endoglucanase enzyme belonging to the glycoside hydrolase family 12 (GH12) and, specifically, hydrolase β-1,4-glycosidic bonds [90]. It is a cellulolytic enzyme that degrades cellulose, enabling the production of biofuels. Cel12A can be delivered with PVX [89], enhancing enzyme stability, reusability and ease of separation from the reaction mixtures [88,91].

Directed enzyme prodrug therapy (DEPT) can enhance the specificity of chemotherapy by targeting enzymes to the tumor site to trigger the activation of prodrugs into cytotoxic substances at that site [92]. Prodrugs are thus administered in a nontoxic form and only converted to the toxic functional form at the target site by targeted enzymes [93]. For example, herpes simplex virus thymidine kinase (HSV1-TK) converts ganciclovir (GCV) into a toxic compound that disrupts DNA synthesis, leading to cancer cell death [94]. Delivery of the HSV-TK enzyme by infectious replication-incompetent herpes simplex virus and administration of GCV have induced cell death through apoptosis rather than direct chemical toxicity and have demonstrated antitumor efficacy in various cancer animal models, including those for glioma, leukemia, bladder, liver, colon and oral cancers [95]. Adenoviral nanoparticles have been used to deliver β-glucosidase, an enzyme that activates the cytotoxic compound irinotecan, specifically within the tumor microenvironment [96]. Similarly, retroviral nanoparticles have been employed to deliver the enzyme purine nucleoside phosphorylase, which converts the prodrug fludarabine into a potent cytotoxic agent [97].

### 3.5. Cancer Vaccines and Immunotherapy

Plant viral nanoparticles are a promising platform for producing cancer vaccines due to their structural characteristics and ability to elicit an immune response [98]. Cancer vaccines are designed to stimulate the immune system to recognize and destroy cancer cells that frequently avoid immune detection due to their analogy to normal cells [99]. VLPs actively engage the host’s immune system, resulting in a longer lasting and specific response [100]. This positions these nanoparticles as an extremely promising platform for long-term cancer management, most especially in the treatment of recurring cancer cells [101]. PVNPs, originating from plant viruses, can be modified to present antigenic structures to induce an immune response [10,102]. Furthermore, PVNPs can stimulate dendritic cells, resulting in a stronger antitumor response when used as a delivery system for tumor-specific antigens [103,104]. Tumor antigens, such as HER2 [105], CEA (carcinoembryonic antigen) [106] or MUC1 [107,108], are frequently overexpressed in certain cancers, making them good targets for therapeutic interventions. Engineering PVNPs to present such peptides/proteins enhances the immune system’s function in recognizing and targeting cancerous cells [104]. Research has revealed that PVNPs that display the HER2 antigen, associated with breast cancer, can elicit a strong immune response, promoting both antibody production and cytotoxic T-cell activity [109,110]. This response is vital to establish long-lasting immunity against tumors, as cytotoxic T cells play an important role in identifying and eliminating cancer cells that express HER2 [111]. Additionally, some PVNPs have been tailored to target melanoma by expressing MRT-1, a T-cell-recognized melanoma antigen, or glycoprotein 100 (gp100) [112]. One preclinical study demonstrated that VLPs displaying these antigens could induce an effective antitumor immune response and significantly inhibit tumor growth [100]. VLPs originating from plant viruses, such as CPMV or TMV, have been modified to display tumor-associated antigens (TAAs) on their surface to stimulate the immune system to target cancer cells [73]. Their repetitive structure enhances antigen presentation and promotes a robust immune response compared to soluble antigens. One animal study has shown that TMV VLPs have a low toxicity [113] and that chimeric TMV-IL-33 VLPs can elicit a strong immune response, resulting in selective targeting and killing of tumor cells [114]. Preclinical research has yielded positive findings, and clinical trials on VLP-based therapeutics for cancer cells are in their early phase [115].

### 3.6. Medical Imaging in Cancer Diagnostics and Therapies

PVNPs also have potential for multimodal medical imaging and therapy. These viral nanoparticles can be used for optical and magnetic resonance imaging by incorporating fluorescent molecules or magnetic nanoparticles, respectively, to monitor the progression of cancer and the efficacy of treatment in real-time [116,117]. These nanoparticles can be modified to carry contrast agents, including iron oxide or fluorescent dyes, for enhancing imaging techniques like magnetic resonance imaging (MRI), computed tomography (CT) and fluorescent imaging, allowing for high-resolution and specific visualization of biological targets [118,119]. For example, Cowpea mosaic virus (CPMV) has been modified to develop imaging agents targeting cancer by incorporating fluorescent dyes, polyethylene glycol (PEG) polymers and targeting ligands to enable selective in vivo tumor imaging [120,121].

Furthermore, PVNPs can be engineered to generate reactive oxygen species upon exposure to specific wavelengths of light, thus being suitable for photodynamic therapy applications [122].

### 3.7. Safety and Regulatory Considerations for PVNPs

One of the advantages of PVNPs is that they are produced in whole plants or plant cell suspension cultures that are free from animal pathogens and toxins [123], rendering them safe for administration to humans and other animals. The ability of glycan moieties on plant-derived glycoproteins to elicit allergic responses in patients [124] has necessitated investigations into the safety profile of plant-derived biologics [125,126]. Plant glycoproteins contain β-1,2-xylose and core α-1,3-fucose structural motifs, which are not found in human glycoproteins [127]. They lack the terminal β1,4-gal and N-acetylmeuraminic acid (Neu5Ac) residues and are usually terminated by mannose residues, such as MMFX or MUFX [127]. In order to elucidate the effect of these plant-specific structural motifs on allergic responses in humans, 280 subjects were enrolled in phase I/II clinical trials testing plant-produced Influenza VLP-based vaccines [128]. Although 40 of the subjects had declared allergies and 23 were allergic to plant material, no allergic responses were initiated or worsened in any of the individuals during the 6-month study. Only weak and transient immunological responses (IgG and IgE) were observed against common plant glycans, and no subject developed IgE responses against the MMFX or MUFX motifs associated with hypersensitivity reactions [129,130]. Indeed, it has previously been reported that a large majority of adverse responses are directed against plant proteins instead of plant-specific glycoepitopes [131,132]. A study tested plant-produced H5N1 virus-like particles (VLPs), a subset of viral nanoparticles, in preclinical and phase I clinical trials and found that they were safe, were well tolerated and did not induce allergic reactions in inoculated humans [133]. A quadrivalent VLP-based vaccine (QVLP) containing influenza A and B VLPs was found to be safe, immunogenic and effective in phase I/II, phase I and phase III clinical trials performed in humans [134,135,136,137]. The plant-expressed influenza VLPs as well as plant-expressed SARS-CoV-2 VLPs [138,139] were recently marketed by Medicago, Inc. [140]. The approval of these products by the relevant regulatory authorities represents a breakthrough in the acceptance of plant-derived biologics and their production systems by regulatory agencies. The use of Papaya mosaic virus VLPs as an adjuvant was also recently tested in a phase I clinical trial and was found to be safe with no adverse side effects observed in vaccinated individuals for 3 years following vaccination [141].

Although not a PVNP, a plant-made therapeutic enzyme treating Gaucher disease was approved by the FDA in 2012 [142]. Indeed, the clinical trial in glucocerebrosidase-deficient Gaucher disease (GD) patients showed no apparent risk to patients when these patients were injected with taliglucerase alfa, a version of human glucocerebrosidase produced in plant cells [143]. Similarly, no adverse effects were observed in a phase I clinical trial where humans were injected with a recombinant glucocerebrosidase enzyme expressed in transformed plant cells, demonstrating safety and a lack of immunogenicity [144].

Most human clinical trial studies using plant-expressed, virus-like particle systems to date have not reported any severe adverse effects. It must be noted that each plant-derived biologic is unique and needs to be assessed individually. The specific plant expression system; reproducibility of production methods; as well as the structure, purity, potency and stability of the biologic itself are investigated during the assessment [123,145].

### 3.8. Technoeconomic Analysis of Plant Biologics

The production costs for PVNPs may be more favorable than those of other nanoparticle manufacturing methods. While downstream purification is a key cost driver contributing significantly to the total cost of production due to stringent clinical grade purity requirements in all production systems [140], plant-based systems eliminate the need for costly bioreactors, thereby reducing the overall production costs compared to, for instance, mammalian cell expression systems [146]. Monoclonal antibodies and their derivatives are currently the dominant class of biologics on the market for human therapeutics [147]. Monoclonal antibodies (mAbs) are utilized in research, diagnostics and therapeutics for various infections and cancer. According to Novaoneadvisor 2024 report #8686 [148], the global market size for mAbs therapeutics is currently USD 233.19 billion in 2024 and is expected to increase to USD 919.06 billion by 2033, with a CAGR of 14.7%. It is not surprising that technoeconomic analysis to assess production costs in plants has focused mainly on this biologics class [149]. A direct comparison of antibody production costs in plants and mammalian cells was conducted by [150]. Whereas a Chinese Hamster ovary (CHO) facility annually producing 250 kg mAb at a 1 g/L yield and a 70% downstream recovery rate had a cost of goods sold (COGS) of $260/g and operational expenses of $65 million/year [151], a transient expression *Nicotiana benthamiana* plant facility of a similar production scale was predicted to have a COGS of $131/g and OPEX of 33 million/year [150]. This represented an approximate reduction of 50% in COGS and OPEX when utilizing a plant production facility. In a mammalian production facility producing 1000 kg of monoclonal antibody annually at a 1 g/L yield, the downstream processing costs were $232/g [152], whereas a *Nicotiana benthamiana* plant production facility with a 600 kg/year capacity was predicted to have a COGS of approximately $99/kg, inclusive of upstream and downstream processing costs [150]. This is a more than 50% reduction in manufacturing costs and indicates the cost advantage of plant-based expression systems compared with mammalian cell-based protein production systems.

Despite the clear advantages of plant-derived biologics, large pharmaceutical companies have been slow to embrace plant expression and restructure their manufacturing processes for plant-based production [150,153].

## 4. Conclusion and Future Prospects

Plant nanoparticles show great potential in cancer biotherapy, offering targeted delivery of therapeutic drugs/molecules resulting in enhanced therapeutic efficacy and reduced adverse side effects. The potential cost benefits of plant production systems, their production speed and scalability as well as the distinctive features of plant viral nanoparticles themselves, including their safety, structures, stability, biocompatibility, inherent immunity and adaptability, position them as a compelling platform for prospective cancer therapeutic applications, resulting in the development of more effective and personalized cancer treatments. Limitations include their perceived ability to include a hypersensitive response in vaccinated individuals, requiring the need for comprehensive clinical evaluation, regulatory approval processes that are not as well defined as those in traditional mammalian expression systems as well as a resistance from large pharmaceutical companies to change their well-established manufacturing protocols [17,154]. As research in this field continues to advance, we anticipate the integration of these innovative nanomedicines into clinical practice, potentially transforming the approach to cancer treatment.

## Data Availability

No new data were generated.
